# Towards biocultural realism: Connecting conservation with historical ecology and common sense. A European perspective

**DOI:** 10.1007/s13280-024-02089-2

**Published:** 2024-11-06

**Authors:** Andrzej Bobiec, Ian D. Rotherham, Simay Kırca, Zsolt Molnár, Mauro Agnoletti

**Affiliations:** 1 Institute of Agricultural Sciences, Land Management, and Environmental Protection, University of Rzeszów, Rzeszów, Poland; 2 Advanced Wellbeing Research Centre, Sheffield Hallam University, Sheffield, UK; 3 Department of Landscape Planning and Design (Current Affiliation), Istanbul University-Cerrahpasa, Istanbul, Turkey; 4 Centre for Ecological Research, Institute of Ecology and Botany, Vácrátót, Hungary; 5 Faculty of Agriculture, University of Florence, Florence, Italy

**Keywords:** Bio-cultural diversity, Conservation failure, Common agricultural policy, Historical ecology, Paradigm change, Subsidiarity

## Abstract

In this perspective, we present and discuss four major causes of the worldwide nature conservation failure: 1) ideologies based on nature–culture dualism, 2) the bias prioritising forests in conservation, 3) the illusory objectiveness of selected biological indicators, and 4) the mismanagement of rural agricultural landscapes. All of these relate to ignorance of historical ecology and neglect of the role past plays in shaping landscapes and fostering biodiversity. These led to a false anthropology focussed on the broader human economy (including agriculture) as the absolute culprit of biodiversity loss. It is believed, therefore, that biodiversity preservation depends on conservation policies and actions providing protection against human activities, such as farming. In this way, nature conservation has been detached from the rich experiences of long and fruitful coexistence of people with other elements of nature. The bio-cultural legacy includes biodiversity-rich rural landscapes, whose habitats are often either neglected or wrongly interpreted as “remnants of natural ecosystems”. Consequently, conservation efforts are frequently ineffective or worse still, counter-effective. In the face of policies favouring subsidised intensive agribusiness at the cost of destroying smallholder family farming, even expensive conservation projects are usually nothing more than a “fig leaf” to cover failure. We advocate re-focussing of conservation planning to put more emphasis on landscapes’ historical ecology responsible for their bio-cultural diversity. It implies the need for new principles in policies necessary to secure the economic and cultural sovereignty of local socio-ecological systems responsible for the world’s bio-cultural diversity.

## Misconceptions in conservation

Long-settled human communities developed diverse arrays of land and water-use practices to manage and protect the natural resources they depend on for their livelihoods. Whether or (usually) not our ancestors consciously protected biodiversity, their overall ways of being, including values, socio-economic systems, and institutions, effectively conserved, and developed living landscapes over millennia. Indeed, until the dawn of the industrial revolution, these socio-economic systems, acting almost akin to a Noah’s Ark, developed and sustained Europe's anthropogenic biomes or anthromes (Ellis and Ramankutty 2008), the key harbours of its biodiversity.

Present day conservation biology developed in response to the extinction crisis ever deepening since the industrial revolution and the changes in agriculture during the nineteenth and twentieth centuries (Ceballos et al. 2015). However, the overall effectiveness of science-based conservation policies remains unsatisfactory (Pimm et al. 2014; Eurostat 2023). In this paper, we present several misconceptions that, in our view, may be culprits of the conservation failure. We argue that most of these problems stem from the way we perceive the human economy, particularly, land use and its effects on biodiversity. The identified misconceptions create erroneous attitudes assuming the dichotomy between human economy and environment. A paradigm shift is required to move away from eco-centrism towards an eco-realistic, inclusive, frame of reference for human communities and their ecological context.

## The paradox of “the world without us”

A. Weisman, in his best-selling “The world without us” (Weisman 2007) speculates on the wonders of a once depopulated Earth. The author refers to contemporary proxies of human-less “pristine” nature, including the Białowieża Forest stretching across the Polish–Belarussian border. In his narrative, however, the author neglects the fact that the trajectory of the revered “natural processes” has been substantially determined by historic factors related to human economy. Another failure of contradicting man and nature is the evolutionary one: would there be any reasonable motivation for a species to care about its natural environment without any wellbeing incentive? That motivation has been replaced by “eco-centrism”, with an authority to attribute sovereign “rights” to nature itself (Borràs 2016). Although it does not provide any rationally sound solution to the logical conundrum (as pointed by Schaubroeck 2018), it offers a sense of moral superiority to “anthropocentrism”, identified as human selfishness and greediness (e.g. Washington et al. 2017; 2018).

Interestingly, the commonly expressed paternalistic praise of indigenous cultures’ respect and care for “Mother Earth”, is usually contrasted with anthropocentric “seeing all of nature as just a resource for human use” (Washington et al. 2018). We find this contrasting idealistic and erroneous. Respecting and caring for nature do not imply an assumption that human life and human interests are of equal importance as lives and needs of other species. For thousands of years, the restraint and frugality, required of people by the three Abrahamic religions, shaped the relationship between humans and nature in large areas of the Mediterranean basin (Okyere-Manu et al. 2022). Denying that may have contributed to modern societies’ alienation from their bio-cultural contexts, triggers further misconceptions. One of them is the widespread belief that restoring human impact-free habitats of “pristine naturalness” will save the planet (see the land-sparing vs. land-sharing debate, e.g. Phalan et al. 2011). This idea implies that the wilder ecosystems are, the more they are capable of ensuring life on Earth for future generations. We argue that human cultures connected to the environment through subsistence traditional land use, rather than planet’s own custody of nature, proved to be efficient biodiversity vehicles, sustaining the Europe-dominating, mostly anthropogenic biomes.

## Misleading baselines and “forest bias”

**Fig. 1:**
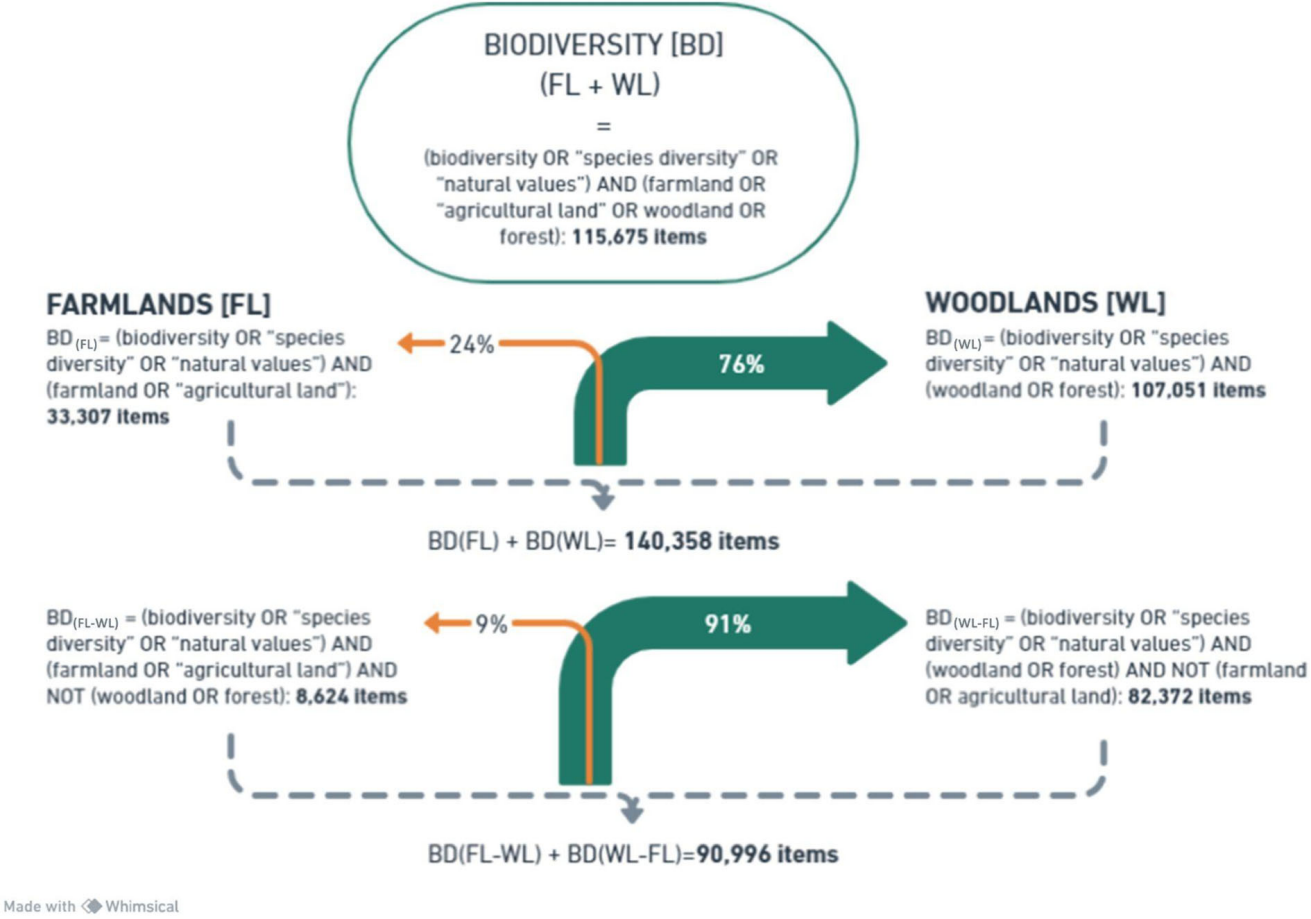
“Forest bias” in biodiversity research shown by the ScienceDirect query (Dec 20, 2023; 20:00 CET)

The most typical naturalness ideal, adopted by modern urban societies is the one of “wild nature”, which in temperate Europe is commonly identified with “primeval”, “virgin”, or “undisturbed” forests. Despite the increasing awareness of the contribution of historical land uses to the diversity of present landscapes (e.g. Rotherham 2014), the appeal to “pristine naturalness” remains a strong motivation for the “hands-off” way of conservation. Therefore, as the most of Europe’s temperate zone, in case of undisturbed ecological succession, would eventually turn to various forest communities (Europe’s “Potential Natural Vegetation”, PNV, Tüxen 1956), most of the conservation debate is focussed on woodlands or forests (Fig. 1).

The “forest bias” has a strong historical and ideological background. Modern forestry was invented and developed in opposition to the former, historical land use, integrating husbandry and woodmanship (Rackham 2008). The collapse of the agro-silvo-pastoral system, leading to segregated specialised agriculture and whole tree timber forestry, was broadly described by Vera (2000). At the very dawn of Europe’s modern forestry, traditional agricultural and pastoral practices were branded as harmful to forests: “The damages done to forests by pasture have been realised a long time ago (…). Therefore, forests should be closed to all livestock forever” (Däzel 1788, after Hölzl 2010). The contrast between open, “degraded” (by cattle and peasants) woods and dense, well-stocked stands, managed by professional foresters, has overwhelmingly been adopted by naturalists, recognising dark, close canopy timber woods as closer to the PNV “ideal” than “degraded” semi-open woods. Such opinions can be found in seminal writings throughout the nineteenth and twentieth centuries (e.g. Brincken 1826; Faliński 1986). Perhaps one of the most conspicuous single “forest bias” examples is the acceptance of pedunculate oak (*Quercus robur* L.) as late-successional forest species (Leuschner and Ellenberg 2017)–quite in contradiction with the species’ life strategy (Bobiec et al. 2018).

On the level of public conservation discourse, the “forest bias” translates to the dominance of forest issues. “Deforestation” and “tree cutting” is seen as the major threat to biodiversity, even by the societies of regions undergoing steady increase in forest cover (Bobiec et al. 2021). Unfortunately, the ubiquitous eradication of Europe's diverse, traditional farmlands which, once abandoned, undergo succession, or are sacrificed to developmental sprawl (Grădinaru et al. 2015) does not evoke public concern comparable to common pro-wilderness zeal. There are numerous examples of the “hands-off” approach wrongly applied to biocultural habitats shaped by historical land uses. For instance, developed under specific zoo-anthropogenic disturbances, biodiversity-rich semi-open woods, once abandoned, undergo succession leading to dense forest ecosystems. Such close-canopy woods are a paradise for shade- and moisture-loving fungi, but they disfavour thermophilous organisms including saproxylic beetles inhabiting old, veteran trees (Horák et al. 2018). Paradoxically, many of such species, e.g. stag beetle (*Lucanus cervus*), hermit beetle (*Osmoderma eremita)*, or great capricorn beetle (*Cerambyx cerdo*), are considered “primeval forest relicts” (Eckelt et al. 2018). That reasoning stems from the “primeval forest” paradigm, according to which the richest contemporary old-growth forests are remnants of a “primeval forest” that used to stretch across the whole temperate Europe, before being altered by man (e.g. Veen et al. 2010; Przepióra and Ciach 2023). However, as the increasing eco-historical evidence shows, much of the revered “primeval forest” traits are the legacies of historical ecosystems, subjected to (zoo-)anthropogenic disturbance regimes (Vera 2000; Bobiec 2012; Horák et al. 2018). As Bergmeier et al. (2010) showed, the commonplace adaptation of the dense “primeval forest” reference to the conservation practice results in an irreversible loss of ecological characteristics that have been sustained by hundreds of years of the traditional use of wooded landscapes.

Finally, with forests being long assumed the most efficient carbon sink, mass tree planting and afforestation have become a global strategy in combatting climate change, propagated and subsidised worldwide, often at the expense of bioculturally rich farmlands and their ecosystem services (Veldman et al. 2015; Agnoletti et al. 2022; Prangel et al. 2023). However, ever-improved modelling provides the increasing evidence, challenging the oversimplified assumption and pointing at uncertainties and the risk of forest transformation into a net carbon source (e.g. Kurz et al. 2008; Williams et al. 2016; Wardlaw 2022).

## Illusory objectiveness of indicators

**Fig. 2:**
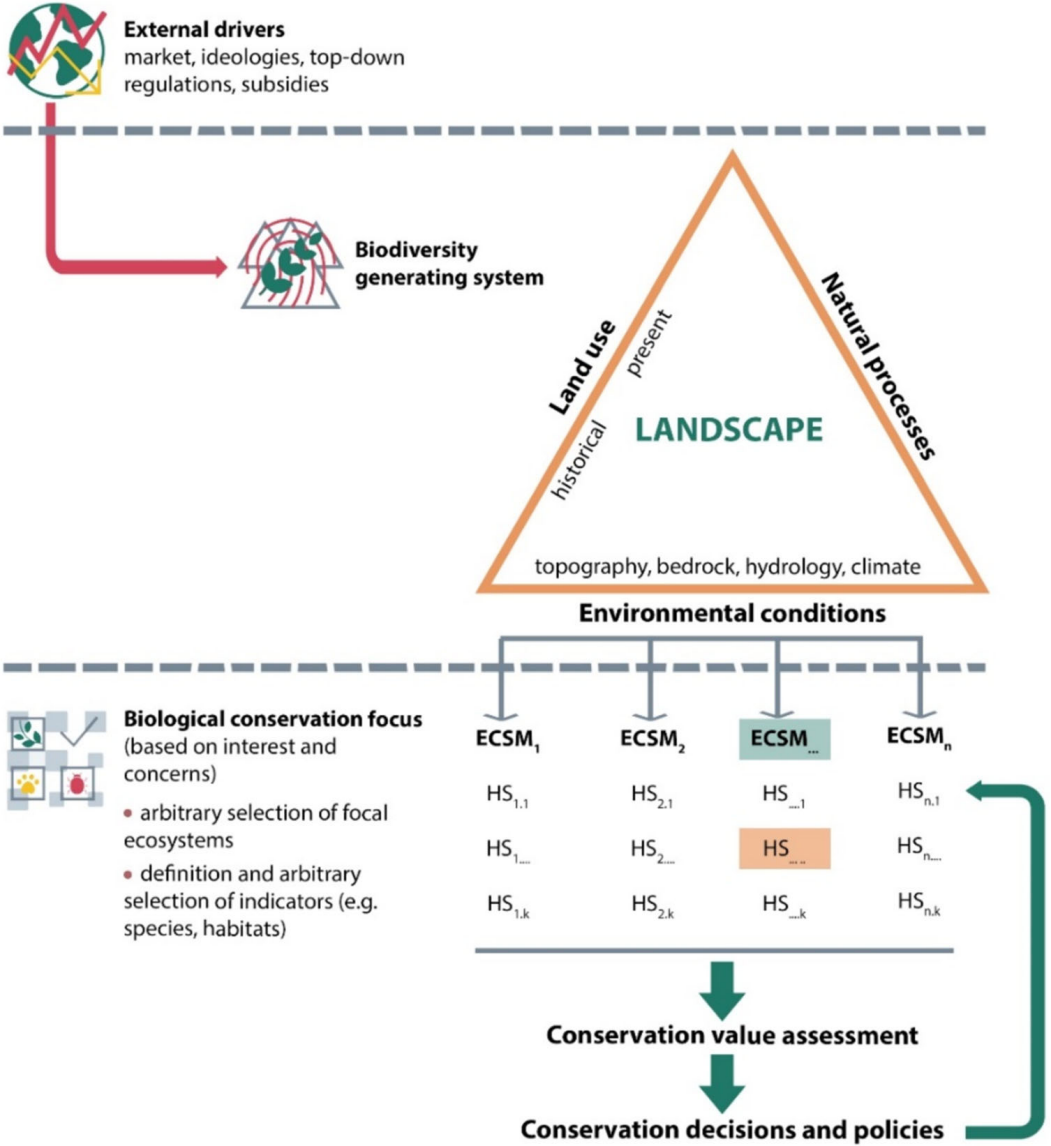
Realm of the biodiversity-generating system, affected by global drivers and often overlooked by the modern conservation biological approach; ECSM–ecosystem, HS–habitat/species, green frame –focal ecosystem(s) selected on the basis of an arbitrary decision, orange frame–arbitrarily selected habitat(s)/species(s), considered as indicators

**Fig. 3:**
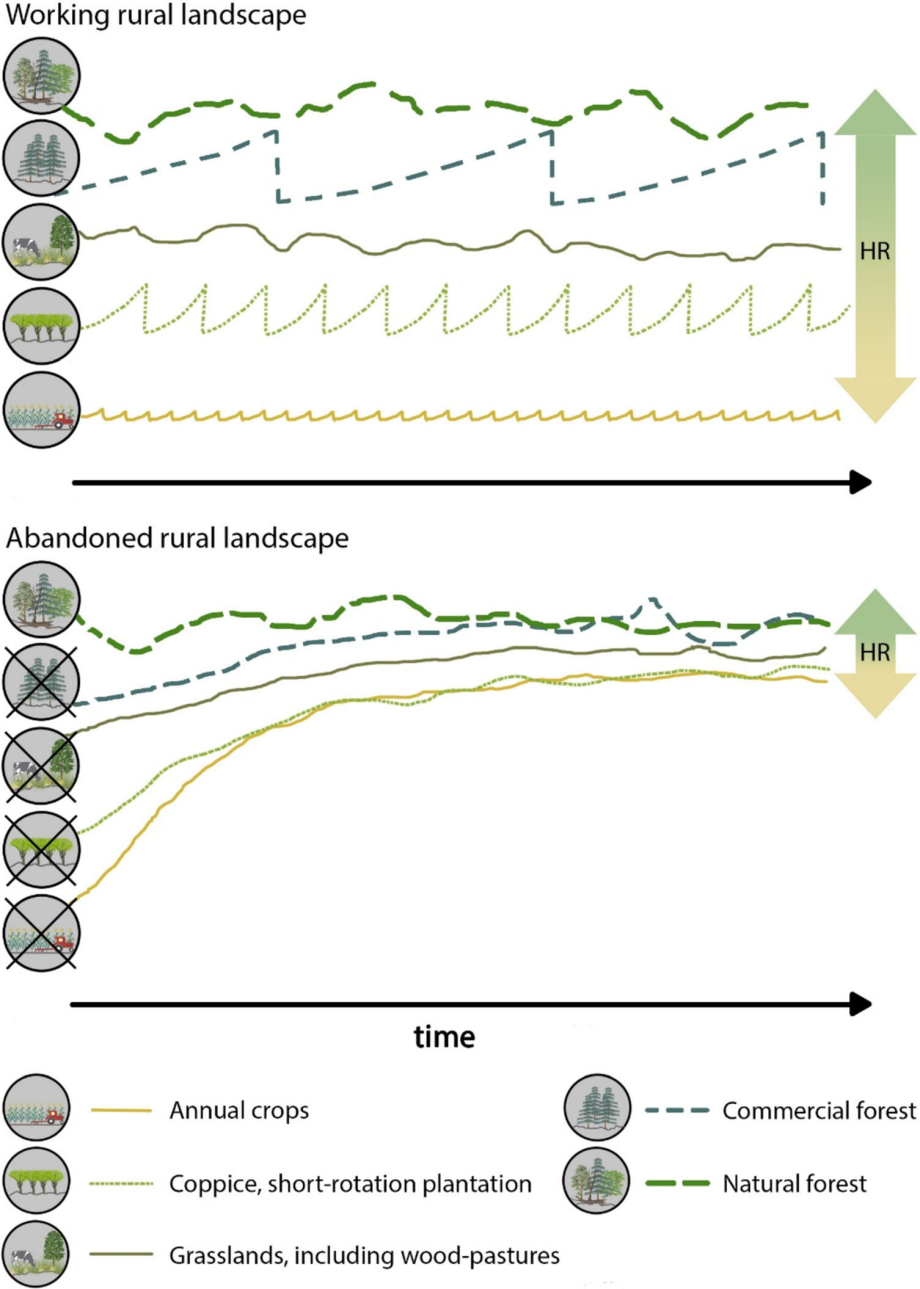
Conceptual diagrams of a working landscape sustained by a system of asynchronic disturbance regimes related to multi-functional farming (upper part); a landscape change corresponding to the ecological succession triggered by land abandonment (lower part). The lengths and heights of regularly oscillating lines symbolically refer, respectively, to frequencies and intensities of anthropogenic disturbances; natural forest dynamics and semi-natural grazing result in irregular fluctuations; while, the landscape’s multiple use maintains the wide range of habitats (HR), producing high gamma diversity (upper part), ecological succession narrows HR, reducing gamma diversity (lower part); graphics were created by using Integration and Application Network; ian.umces.edu/media-library

Misinterpreting the species associated with old veteran trees as indicators of “primeval forests” is not the only pitfall while assessing conservation values of ecosystems. With progressing research in conservation biology, defined as “the applied science of maintaining the earth’s biological diversity” (Hunter Jr and Gibbs 2007), one expects that it will provide an objective base for best conservation practices. Due to complex interactions of natural and cultural factors determining the development, composition, and dynamics of landscapes and their particular habitats, scientific conservation refers to selected, specific but discrete data, considered “biological indicators”, including “indicator species” (Fig. 2). Despite the concept’s simplicity and its great appeal to conservationists, concerns were raised about potential downsides of the “indicator species” use in conservation practice (Simberloff 1998; Büchs 2003). Perhaps the most common indicator approach, based on taxonomic criteria, are the ones of The Birds Directive with its Annex I (ECC 1979) and the Habitat Directive with its Annexes II, IV, V (ECC 1992), two pillars of the European Union’s conservation system. The annexes consist of the lists of species (mammals, birds, invertebrates, plants, and fungi) defined as the “species of Community, i.e. EU’s, interest”. The “species of interest” are assigned “conservation status” such as “rare”, “endangered”, or “vulnerable”). However, the selection of such focal species or/and habitats, despite being arbitrary, is affected by various methodological constraints, leading to biased diagnoses of ecosystems’ states and processes (e.g. Willie et al. 2012). This is because the biodiversity-generating system in reality is not a single, even highly diverse ecosystem, but the whole landscape, depending both on natural environmental conditions as well as on the past and present land use, triggering the landscape-specific natural processes (Fig. 2). In Europe, the traditional, integrated land use, involving animal husbandry and woodmanship, has long been a decisive driver, creating and sustaining multiple habitats, patterns, and disturbance regimes absent in “pristine nature” (Poschlod and Bonn 1998; Vera 2000; Rackham 2008; Agnoletti 2014; Agnoletti et al. 2022). Therefore, such traditional, multi-functional land uses, generating and sustaining additional landscape scale heterogeneities and dynamics, substantially contribute to Whittaker’s (1960) “gamma diversity” (Fig. 3). Just as at the very local scale, botanists’ intentional efforts result in very high diversity in botanical gardens, farming systems based on variegated smallholder farms are analogous, though unintentional biodiversity aggregators and amplifiers at a broader, landscape scale. Their abandonment triggers successional processes leading post-agricultural habitats asymptotically to a common “quasi-climax” PNV state, deprived of the farmland-specific landscape diversity (Fig. 3). Despite the large amount of evidence of the smallholder farming importance for farmland biodiversity (Benton et al. 2003), human economic activity is usually considered a nuisance; therefore, conservation decisions are expected to minimise the human impact (Agnoletti 2014; Agnoletti et al. 2022).

We suggest the that ill-assumption of the objectiveness and universalism of ecological indicators has become an important building block of the system, imposing globally invented solutions on local land use systems which developed through long-lasting interactions of human communities with their immediate environmental context. Considering the high complexity of semi-natural agricultural landscapes and the difficult interpretation of species’ responses to complex environmental variables this assumption may easily lead to local conservation failures. Following his systematic review on ecological indicators, Büchs (2003) warned that it would be impossible to deliver the easy-to-use bioindicators as expected by “spoiled” technocrats. Twenty years later, numerous “surrogate” indicators (like length of hedgerows, field margins, pesticide and fertiliser input), seem to meet such expectations, as shown by the increasing popularity of various measures among experts and policy makers, such as Intergovernmental Science-Policy Platform on Biodiversity and Ecosystem Services (IPBES, i.e. Faith 2021). However, we are sceptical about whether and how these novel concepts (e.g. “non-carbon benefits”, “carbon farming”), quickly replaced by newer ones, can actually increase the conservation effectiveness.

Simultaneously, the painstaking bureaucratic procedures and the very technical, hermetic conservation jargon referring to the assessment of specific indicators, provide a strong advantage to experts and conservationists over local land users–the true stewards of landscape’s biocultural diversity (e.g. Lovrić et al. 2018). Although public participation has become mandatory in most decision-making protocols, we point to the unequal power of parties involved in conservation planning and decisions. While professionals provide ready-to-use solutions, local communities are those to be “educated” and persuaded to provide their expected agreement (Kati et al. 2015; Ece et al. 2017; Strzelecka et al. 2021). Such a condescending sense of the mission of conservation stems from the above-discussed nature–culture dualism, assuming agriculture to be among the major culprits behind biodiversity loss (Chaudhary et al. 2018). This would be justified if only applied to intensive, industrial farming, which, however, developed at the expense of traditional–biodiversity-friendly–agriculture (Barthel et al. 2013; Agnoletti and Santoro 2022).

## Agriculture: Neglecting and destroying sources of diversity

The Enlightenment’s belief in the progress powered by science and technology has affected almost all aspects of human activity, including land use. To boost farming efficiency, modern agriculture has been harnessing any new inventions helping to meet that goal, including artificial fertilisation, pesticides, machinery, and genetically modified organisms. That modernisation has been achieved at the cost of the loss of traditional farming, stigmatised as inefficient and backward, as an impediment for economic development (Heidhues and Brüntrup 2003). With such prejudice, increasingly urban and industrialised societies, disconnected from farmland’s nature, readily adopted radically reformative approaches to farming, best represented by the Rockefeller Foundation’s philanthropic programme (Nally and Taylor 2015). According to its global agenda, the elimination of subsistence, traditional husbandry has become an indispensable condition of the “conquest of hunger” (“Throughout the world, traditional or subsistence agriculture can and must be replaced with a highly productive, market-oriented system”–The Rockefeller Foundation 1968).

The apparent “crusade” against traditional, family-based, farming has become a common denominator of various modern ideologies, economic systems, and political regimes, ranging from capitalistic liberalism to Stalinist communism, and to present-day globalism, as exercised in various parts of the world (Wender 2011). The new wave of “agricultural improvement” could have been observed in post-Soviet European countries adopting global market rules and European farming policies. In Poland, for instance, traditional small family farms successfully survived multiple communism-driven disadvantages and impediments, as the nation’s major food supplier and the most reliable and enduring land steward for almost half a century. Paradoxically, it was not the communist regime but the early 1990s’ liberal agenda that led the traditional, family-based farming to the brink of annihilation (Halamska 2016). The further loss of ca. 0.5 M Polish farms took place during the first sixteen years of subordination of Poland’s farming to the centralistic, subsidy-based Common Agricultural Policy (CAP) of the European Union (GUS 2020; Bilewicz and Bukraba-Rylska 2021).

**Fig. 4:**
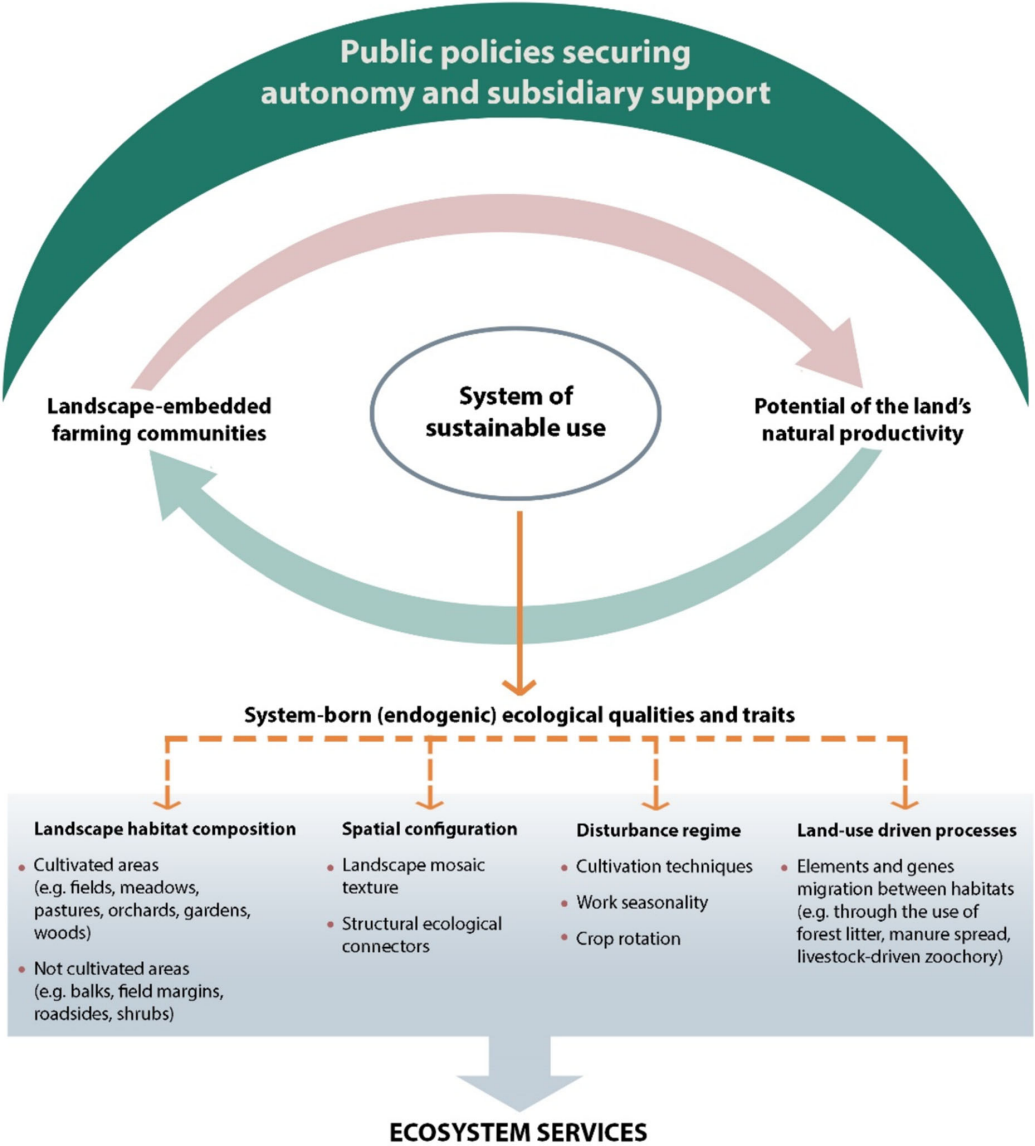
Protecting and supporting autonomous socio-economic-ecological systems of smallholder family farming as a the most effective public investment in biocultural diversity and its ecosystem services

With no reference in the imaginarium of “wilderness” – so appealing to city dwellers – farmlands are far more prone to the erosion of their natural values than woodlands (see e.g. the Common Bird Index). This is despite farmland being the major locale for the studies and inspiration of numerous great naturalists, including Carl Linnaeus. Concentrating on “wild nature”, we allowed sustainable family farming to be subjected to ruthless forces of global market and policy agendas disempowering peasant farmers, leading to landscapes’ cultural severance (Rotherham 2013), and destroying socio-ecologically integrated village systems (Morris and Kirwan 2011). As Chapman et al. (2019) show, various conservation incentive programmes dedicated to rural communities fail due to the conservationists’ ignorance or disrespect for farmers’ values – essential for the integrity of the socio-economic-ecological framework of farming communities (Figs. 2, 3 and 4).

## Re-focussing conservation

We have presented four areas in which the practice of nature conservation is inconsistent with its very fundamental purpose. Philosophically (and logically) questionable perception of natural values, “forest bias”, illusory objectiveness of indicator-based approaches, and neglect of the role of agriculture as an amplifier of biodiversity, appear to be the “cardinal” sins of the modern conservation system(s). Considering these, we imply that conservation policies focussed on biodiversity as an inherent value, separate from human economy, are doomed to fail. Therefore, acknowledging the broader context of human values, socio-economic systems should be allowed to reconnect with their local cultural and ecological contexts as represented by traditional farmlands. This is the best way to optimise the use of natural landscapes’ productivity, lessen dependence on industrial farming, and sustain various ecosystem services provisioned by traditionally farmed landscapes. In order to restore such embedment, we advise adopting the following principles: of (1) historical ecology approaches to natural values, of (2) sovereignty and clarity of conservation-related decisions, and of (3) subsidiarity-based instead of subsidy-based support of nature-friendly land use.

### The role of historical ecology is unlike “classical” ecology which over-dominantly considers presently observed processes with their immediate causes and consequences

Historical ecology addresses the effects of historic human impacts on organisms (e.g. pollarded trees), habitats (e.g. through prescribed burning, grazing, ploughing), landscapes (through specific land use structure), and whole regions (through specific economic-historical models). The historical ecology approach allows a more focussed interpretation of observed objects, systems, and processes. Is the current situation a representative “snapshot” of long-lasting, natural “dynamic equilibrium”, or a result of new dynamics, overlapping historical development? If the latter is the case, then how advanced is the current dynamic? What is the degree of change since the shift in the disturbance regime?

Neither ubiquitous spread of woody vegetation on abandoned fields, nor wilding of ungrazed, semi-open groves and woods, should be interpreted as straightforward evidence of the ecosystem’s recovery after removal of ecologically degrading anthropogenic factors. Perhaps, the best “rule of thumb” one should use while adapting an optimal frame of reference, is a relative duration of a specific disturbance regime, crucial to the development and sustaining of the recognised present biological or bio-cultural values. If it was an anthropogenic disturbance regime, natural, spontaneous development would certainly not preserve the *status quo*. Therefore, one should decide whether to: (a) sustain the long-lasting and still functioning disturbance regime responsible for the present condition of the object (i.e. a habitat or a landscape); (b) restore the original state of things as developed under the historical (past) disturbance regime; (c) halt further directional development at a present stage (e.g. through introducing grazing on abandoned feral farmland); (d) re-design the shape and functioning of the object through novel solutions of land management; (e) sacrifice the *status quo* to open-ended, continuous wilding projects (Fig. 3, bottom part). Considering the huge scale of agricultural abandonment and the following spontaneous landscape change (Schulp 2019; Debonne et al. 2022), informed management/conservation solutions are applied mostly in isolated cases.

### Sovereignty and clarity of conservation decisions

The final consequences of any conservation decision are subject to uncertainties. The more detailed, quantitative definition a conservation objective has, the higher the risk is of a development diverging from that assumed objective. Natural and bio-cultural systems perform as multi-level entities, consisting of abiotic and biotic components, interconnected through various types of interactions. Such structurally and compositionally intricate and dynamically asynchronous systems are additionally subject to continuous environmental changes (e.g. Bobiec 2012). Thus, any set of detailed scientific data (e.g. on species composition, population structure, ecological indicators) is to a degree, an arbitrarily selected representation of the system’s immediate, present status, with very limited potential of reliable generalisations or extrapolations.

Narrowly defined conservation targets, due to the species’ mutually exclusive niches and life strategies, may result in low conservation effectiveness or even lead to degradation of protected objects (e.g. Britnell et al. 2021). Among numerous examples of discrepancies between initial conservation objectives/expectations and actual effects, are former silvo-pastoral, semi-open woods erroneously interpreted and managed as “forest habitats” (e.g. Bergmeier et al. 2010). Inadequate definition of the ecological niches of “target” species in protected areas means inappropriate conservation measures placing their populations in peril (e.g. Litkowiec et al. 2018).

We advise that simple, clear justification of conservation initiatives, with explicit, obvious objectives can be far more convincing to the general public. These would generate wider support than scientific, sophisticated protocols readable only to well-trained experts and technocrats. This advice is substantiated by two, though very different, successful approaches to conservation. The first one is focussed on the preservation of “wilderness” and the second one, on the conservation of countryside cultural landscapes. While the concept of “wilderness”, first implemented in the mid-1800s has become an essential part of the US national cultural identity (Nash 1970), culturally shaped countryside has occupied a similarly important position in the sense of “Englishness” or “Britishness” (Lowenthal and Prince 1964). For wilderness, as demonstrated by the history of the New York State Adirondack Park (Terrie 1994), the future value of “untrammelled by man” wild nature, regardless of its biological specification, is much more important than the area’s *status quo* at the starting point. This is the open-ended approach, now building public support for wilderness to benefit future generations. In the case of the historic countryside, its value corresponds with the state of preservation of original features developed and sustained by long-lasting traditional land uses (Rackham 1987). In both examples, though very different, the straightforward conservation message, referring either to common affection for the country’s “frontiers”, or to the respect for inherited historical landscapes, translates into the involvement of both local communities and multi-million membership, powerful NGOs, such as Sierra Club, the National Trust, or the Woodland Trust.

### Subsidiarity—not subsidy-based support for nature-friendly land use

According to the Oxford English Dictionary, subsidiarity is "the principle that a central authority should have a subsidiary function, performing only those tasks which cannot be performed at a more local level". Regarding the undeniable contribution of traditional, self-sustained, and multi-functional farming to biodiversity and functioning of human communities (Molnár et al. 2015), this should be an important beneficiary of potential subsidiary support by central authorities, such as states or international organisations.

The European Union has explicitly adopted the “Subsidiarity Principle” in Article 5(3) of the Treaty on European Union. In practice however, the way it is implemented depends on what, at the EU level, is defined as a priority (European Parliament 2023). An accepted priority of the Common Agricultural Policy (CAP), has meant that the EU administration developed mechanisms aimed to implement the CAP throughout Europe's farming community. The CAP priorities (e.g. favoured crops, milk quota, use of fertiliser and pesticides, climate issues), incorporated in the EU seven-year budget are exerted on farmers through top-down policies empowered with heavy financial incentives or deterrents, undermining cohesion and self-sufficiency of smallholder agriculture. As shown by Scown et al. (2020), the CAP subsidies neither contribute to ecological sustainability nor to higher economic efficiency. The negative role of subsidies, disfavouring self-sustaining smallholder farming and adversely affecting biodiversity is reported from many geographical regions (e.g. FAO 2017).

The net-positive contributions of multi-functional, family, smallholder, and subsistence farming models to bio-cultural heritage are widely acknowledged (e.g. Halada et al. 2011). Self-sustaining, these systems foster and steward complex and diverse landscapes and habitats dependent on specific disturbance regimes secured by land use practices. Conventional approaches to nature conservation through set-aside protected areas are easiest to implement through top-down policies (e.g. Müller 2014). In contrast to this, development and sustenance of spatially, ecologically, and socially embedded farming (Morris and Kirwan 2011) requires respect for its autonomy (Szumelda 2019). Such an approach has been recommended by FAO Globally Important Agricultural Heritage System (GIAHS) programme (Agnoletti and Santoro 2022).

The biological values of working landscapes (as “unintended by-products” of traditional land use) (Vos and Meekes 1999) may seem the most important asset of multi-functional, self-sustained husbandry. However, we anticipate increased appreciation of wider ecosystem services in a time of growing social and economic uncertainty. Autonomous, small, family farms and their communities secure the diversity of local food systems and are thus important factors of food sovereignty (Pimbert 2019) and indispensable sources of high nutritional quality (e.g. Provenza et al. 2015). Interaction between culture and natural environment creates unique landscapes that cannot be preserved and managed simply by reference to biologically specific, globally established criteria and indicators. Therefore, landscape approaches entail more opportunities to address the factors contributing to the current state of global ecosystems. This is especially true in rural areas.

Even though diversified family farming proves its high resilience and adaptability potential (e.g. Grigorescu et al. 2022), liberal deregulation alone with cuts in financial subsidies feeding industrial agribusiness-supermarket network alliances, would be insufficient to establish conditions, for smallholder husbandry to revive and spread (Béné 2022). To reverse the impacts of long-established policies disfavouring traditional agriculture (Bilewicz and Bukraba-Rylska 2021), active involvement of states and regulatory powers underpinning economic justice is required to allow the re-establishment of traditional agrarian practices (Shucksmith and Rønningen 2011). This would be a much-needed form of subsidiarity. Furthermore, a fair and equitable involvement of small-scale land users is needed to bring in their intimate understanding of nature and human-nature relationships into conservation planning. This should be accompanied by an intensive process of knowledge partnership and knowledge co-production where scientific knowledge and local, traditional knowledge can re-focus our ecological knowledge base of conservation, preferably in community-based conservation actions (Molnár and Babai 2021; Molnár et al. 2024).

The dominance of intensive, industrialised farming has long remained unchallenged. Unchallenged, despite its inefficiencies in terms of energy (petrochemical-subsidised machinery and fertilisers), in terms of environmental quality (degradation of soil and pollution of water), in terms of fractured communities and rural depopulation. This is also evidenced by the widespread extinction of biodiversity (including many formerly common species), and compounded by huge problems of economic dysfunction. There is now talk of regenerative farming, and of finding new ways to deliver sustainable good-quality, healthy food without sacrificing the planetary systems. In the meantime, we paper over the cracks with talk of rural landscapes being supported by leisure and tourism for example. Yet the countryside that tourists wish to visit, and the people they wish to meet, are born of traditional land management delivering multiple benefits. Furthermore, with increasing awareness of wider ecosystem services such as flood reduction, climate change proofing, and carbon capture, it is the traditional countryside which delivers these in abundance.

Therefore, we advise a serious worldwide, interdisciplinary debate on the social and economic rehabilitation of smallholder, family farming, including subsistence traditional agriculture, for their role in the provision of multiple ecosystem services to the wider public. We strongly support considering such farms as “biocultural refugia” – indispensable for diversity of practices, for food security and biodiversity (Barthel et al. 2013) – of ever-increasing importance towards the uncertain future. Unlike the market-oriented agrobusiness, (semi)subsistence smallholder farming – as acknowledged by farmers themselves – is not considered a money-making venture, but the very way of life in harmony with nature and tradition, often at a price of any major economic gain (McCarthy et al. 2022). Therefore, with such a minimal focus on profit, traditional (semi)subsistence farming as a whole should be regarded as self-sustained *pro publico bono* activity, contributing not only to biodiversity, but also to public food security, cultural richness, and the general quality of life. Comparing to project-based, entirely dependent on public subsidies, conservation solutions, sustainable, traditional land use provides a far more reliable framework for lasting biodiversity (Sayer et al. 2013). As Henle et al. (2008) identified agricultural change – such as intensification, land abandonment, and up-scaling agricultural operations – as the biggest threats to terrestrial biodiversity, the major task of conservation is to stop and, wherever possible, reverse those changes, which have not evolved from farming communities, but which have been enforced by external pressures. Thus, instead of stigmatising agriculture, patronising farmers to exert on them green agendas, we should establish an effective “umbrella” or buffer, protecting the autonomy of all remaining smallholder, (semi)subsistence family farms and provide any subsidiary assistance they need to keep on going (Fig. 4). At the same time, one should develop reliable re-agrarianisation (Hebinck 2018) policies, providing conducive socio-economic conditions to all who would like to make living out of smallholder farming. Across Europe, there are local attempts to restore farming and prevent the further loss of productive landscapes to wild vegetation, such as the restoration of silvo-pastoral mountain landscapes in the Italian Apennines (Fiore et al. 2024), grazing-led recultivation of the feral post-agricultural landscape in the Polish Carpathian foothills (Bobiec et al. 2024), or ecological intensification of dehesa a multi-functional, agro-silvo-pastoral, cultural landscape of southern and central Spain and southern Portugal (Pardo et al. 2023). However, without wider social and economic climate, favourable to re-agrarianisation, such initiatives will remain merely, isolated exceptions to progressing loss of Europe’s working rural landscapes. As Horlings and Marsden (2011) put it, making a vibrant part of the future land use mainstream out of such isolated remnants of traditional agriculture and new good husbandry practices, would mean a “real green revolution”.

Last but not least, any local or regional improvement of conservation or, more generally, “green” standards, should not be achieved through policies outsourcing problems to far world’s regions and their peoples. Unfortunately, various environmentally–“ambitious” programmes (such as CAP or carbon neutrality), involving gigantic subsidies as well as legal, financial or trade sanctions, contribute to the destruction of (semi)subsistence farming, both in areas of their direct implementation, but also farther away, due to unfair trading conditions. According to Kortleve et al. (2024), more than 80% of the CAP supports intensive animal production, which, together with extremely demanding “animal welfare” standards, make traditional biodiversity-supporting husbandry, both within and outside EU, unsustainable. As Kolinjivadi et al. (2023) observed, much of the “green economy” – so emblematic for the modern, mostly urban societies – considering the over-simplistic understanding of a “green ideal” and the way it is being forced on communities, is simply a continuation of “plantation ecology”, a kind of colonial behaviour, disregarding farmers and detrimental to their landscapes.

## Concluding remarks

In this paper, we discussed how the widely agreed-upon failure to prevent biodiversity loss may have several, often overlooked causes. These include 1) how we understand humanity’s place in nature (and the condition of “nature without us”), 2) the bias towards forests in conservation prioritisation, 3) the illusory objectiveness of selected indicator sets, and 4) the mismanagement of rural agricultural landscapes across Europe.

We suggest re-focussing of conservation planning to put more emphasis on understanding the origins and development of natural values. This requires replacing the present, antagonistic paradigm of nature–culture dualism with bio-cultural realism, considering a positive contribution of human land use to biodiversity. Such a paradigm change is particularly important, whenever cultural landscapes (including most of European landscapes), are at stake. It should involve the historical ecology approach, increased sovereignty and clarity in conservation decisions, and replacement of subsidy-based support mechanisms of nature-friendly land use by respect to the autonomy of local socio-economic systems, based on sustainable use of landscapes’ natural potential. Their further survival and necessary re-establishment of the landscape-embedded husbandry require effective buffering of destructive top-down pressures, combined with subsidiarity.

We advise a serious worldwide, interdisciplinary debate on a social and economic rehabilitation of smallholder, family farming, including subsistence traditional agriculture, for their role in the provision of multiple ecosystem services to the wider public. Such a debate, involving biologists and conservationists, anthropologists, economists, and philosophers, and especially small-scale farmers themselves, should translate to the new social, political, and economic climate fostering restoration and development of the landscape-based sustainable smallholder farming. Replacing the present inefficient, quasi-authoritarian, top-down model of exerting “green” solutions with trust, restraint, and friendly subsidiarity would unleash the great potential of responsible land stewardship, provided by self-sustaining smallholder family farms.

Future conservation science should be based on a holistic approach, integrating social/cultural and natural aspects of our environment. This can be dealt with multi- and transdisciplinary research, involving, among others, biology, anthropology, history, economy, and sociology, as well as the small-scale farming communities. With active participation of land use practitioners, the transdisciplinary conservation may develop a strong “adaptive learning” component, necessary to secure a better understanding of findings and a more knowledgeable and responsible development of land management practices.
